# Using financial incentives to increase initial uptake and completion of HPV vaccinations: protocol for a randomised controlled trial

**DOI:** 10.1186/1472-6963-12-301

**Published:** 2012-09-04

**Authors:** Eleni Mantzari, Florian Vogt, Theresa M Marteau

**Affiliations:** 1 Health Psychology Section, Department of Psychology, King’s College London, Guy’s Campus, 5th floor Bermondsey Wing, SE1 9RT, London, UK

**Keywords:** HPV, Human papilloma virus, HPV vaccinations, Financial incentives, Vouchers

## Abstract

**Background:**

HPV vaccination reduces the risk of cervical cancer. Uptake however, of the ‘catch-up’ campaign in England for 17-18 year old girls is below the 80% NHS target**.** The aim of this randomized controlled trial is to assess the impact of financial incentives on (a) the uptake and completion of an HPV vaccination programme and (b) the quality of the decisions to undertake the vaccination.

**Method/Design:**

One thousand (n = 1000) 16-18 year-old girls will be invited to participate in an HPV vaccination programme: Five-hundred (n = 500) will have received a previous invitation to get vaccinated but will have failed to do so (previous non-attenders) and 500 will not have previously received an invitation (first-time invitees). Girls will be randomly selected from eligible participants who are registered with a GP in areas covered by the Birmingham East and North (BEN) and Heart of Birmingham Primary Care Trusts. The two samples of girls will be randomised to receive either a standard vaccination invitation letter or an invitation letter including the offer of vouchers worth £45 for receiving three vaccinations. Girls will also complete a questionnaire to assess the quality of their decisions to be vaccinated. The primary outcome will be uptake of the 1^st^ and 3^rd^ vaccinations. The secondary outcome will be the quality of the decisions to undertake the vaccination, measured by assessing attitudes towards and knowledge of the HPV vaccination.

**Discussion:**

The key results will be: a) the effectiveness of financial incentives in increasing uptake of the 1^st^ and 3^rd^ vaccinations**;** b) the role of participants’ socio-economic status in the moderation of the impact of incentives on uptake; and c) the impact of incentives on the quality of decisions to undertake the HPV vaccinations.

## Background

Human Papillomavirus (HPV) is an ubiquitous sexually transmitted virus that could lead to cervical cancer [1,2]. HPV vaccines help prevent infection by some of the most common forms of HPV that are associated with later development of cancer [3,4]. The HPV immunisation process takes six elapsed months and is conducted in three stages: 1^st^ vaccine, 2^nd^ vaccine two months later, and a 3^rd^ vaccine six months after the first vaccination. Completion of all three vaccinations is necessary to effectively reduce the risk of cervical cancer [5]. The degree of protection afforded by incomplete immunisation is currently unknown [6].

Since September 2008 a national programme has started in England and Wales aiming to vaccinate girls aged 12-13 against HPV. A two-year ‘catch-up’ campaign that offers the HPV vaccine to 17-18 year old girls has also been initiated. The objective of these HPV vaccination programmes is to provide three doses of the HPV vaccine to females before they become sexually active, when the risk of HPV infection and subsequent cervical cancer development increases. It is estimated that if this objective is met and vaccination coverage is sufficiently high (80% of the target population), up to 400 deaths per year in England could be prevented [7]. Although the national programme in England aimed at 12-13 year-old girls has resulted in high uptake (88.1% uptake of the first vaccination and 80.1% of the third vaccination), the uptake rates for the “catch-up” campaign in England (targeting 17-18 year olds) have been lower, with 62.2% of the target group receiving the first dose and 31.8% the third [7].

Offering girls financial incentives to undergo the HPV vaccination could increase these uptake rates. Incentive mechanisms are increasingly being considered and used in health care policy in the UK and elsewhere in an attempt to change health-related behaviour [8,9]. They are most effective in changing ‘simple’, ‘one-off behaviours’ such as getting vaccinated [10-12]. Their effectiveness however, has been predicted to vary with recipients’ level of social deprivation. Specifically, it has been argued that the most socially deprived should respond more to financial incentives [13]. Most of the calls, to use incentives in HPV vaccination programmes in the UK have so far focused on incentivising those providing the vaccination (e.g. GPs) rather than vaccination recipients [14]. Their effectiveness therefore in this context is currently unknown. Furthermore, no studies have assessed the role of social deprivation in the moderation of their impact on vaccination uptake.

Even if effective in improving uptake of the HPV national vaccination programme, the use of financial incentives raises concerns about the possible adverse effects they may have on the quality of people’s decisions to engage in incentivised behaviours. For example, it has been argued that the prospect of receiving a financial reward could result in the risks associated with a particular health behaviour being overlooked [15].To date, however, no known studies have assessed the mechanisms by which financial incentives influence the decision-making processes involved in engaging in an incentivised health behaviour.

In summary, further research is needed to determine the impact of financial incentives upon first, uptake of the HPV vaccination, and second, the quality of recipients’ decisions to get vaccinated. Furthermore, research is needed to determine the role of social deprivation in the moderation of the impact of financial incentives on uptake of vaccinations.

### Objectives and hypotheses

The primary objectives of the present study are:

(a) To assess the impact of financial incentives on the initial uptake (uptake of the first vaccination) and completion rates (uptake of the third vaccination) of an HPV vaccination programme.

(b) To assess the impact of financial incentives on the quality of the decision to be vaccinated as measured by attitudes towards and knowledge of the vaccination.

The secondary objective is:

(a) To assess whether the impact of financial incentives on the initial uptake and completion rates of an HPV vaccination programme is moderated by participants’ levels of social deprivation.

### Hypothesis I

Those offered financial incentives to get vaccinated against HPV are more likely to receive the first and third HPV vaccinations.

### Hypothesis II

The effect of incentives on uptake of the first and third vaccinations will be moderated by participants’ levels of social deprivation, with larger effects of the incentives being observed for the most socially deprived.

### Hypothesis III

Offering financial incentives reduces the quality of decisions to get vaccinated against HPV.

## Methods/Design

### Trial design

This is a randomised controlled trial in which two independent samples of participants are separately randomised to the offer of financial incentives for getting vaccinated.

### Participants

Participants will compromise of 16-18 year old girls, living in Birmingham. To be included in the trial, girls must fulfill the following inclusion criteria:

(a) Live in areas falling under the administration of the Birmingham East and North (BEN) and the Heart of Birmingham Primary Care Trusts

(b) Be registered with a GP within one of the two PCTs

(c) Be eligible to be vaccinated through the clinics (Sutton Cottage, Partners in Health and Dove Medical Centre)

(d) Not have been vaccinated against HPV before.

Half of the sample will consist of girls who have previously received an invitation to get vaccinated, but have failed to attend the first appointment (previous non-attenders). The remaining half will consist of girls who have not yet received an invitation to attend the vaccination programme (first-time invitees).

### Intervention

The components of the intervention used in the present HPV vaccination programme are:

### Invitation letters

All participants will receive letters inviting them to attend their first HPV vaccination session. These will be sent on behalf of the Birmingham East and North and Heart of Birmingham Primary Care Trusts, and will include the date, time and location of the allocated appointments.

### Reminder text messages

Participants attending their first vaccination appointment will be asked to inform the researchers of their mobile phone numbers. These will be used to send text messages reminding them of their subsequent vaccination appointments. These will be sent during the intervals between the first and second vaccinations and the second and third vaccinations and two days prior to the next session. An example of the wording of these messages is: *“(Name), don’t forget your HPV jab today at (time) at the (venue). Thank you”*.

### Offer of financial incentives

Participants from the two samples (i.e. previous-non attenders and first-time invitees) allocated to the incentivised groups will receive a modified version of the standard vaccination invitation letter, described above, which will include the offer of vouchers worth £45 for receiving the three vaccinations. Specifically, participants will be informed that they will receive:

£20 for the first vaccination

£5 for the second vaccination

£20 for the third vaccination

### Procedure

The trial will be run by the Birmingham East and North Primary Care Trust in collaboration with Healthy Incentives (http://www.healthyincentives.org.uk/, a social enterprise arising as a result of a partnership between the Young Foundation and the Birmingham East and North Primary Care Trust). The Birmingham East and North PCT has employed the Birmingham Primary Care Shared Services Agency (BPCSSA) to do the following: select participants to be included in the trial; randomise them to each group and post the invitation letters. Once the letters have been sent, the BPCSSA will provide the Healthy Incentives team with the details of all the participants who have been invited, including their names, addresses, scheduled vaccination dates, the participant group (previous non-attender or previously not invited) and randomisation group (incentive or not). The vaccinations for all individuals will take place at three community clinics. The BPCSSA will schedule a number of ‘incentivised only’ sessions at these clinics to avoid any tensions caused by not incentivising all groups. Vaccinations will be carried out by nurses working with Heart of Birmingham (HOB). When attending their first vaccination session and while waiting to get vaccinated, participants will be asked to sign a consent form and complete a measure assessing the quality of their decision to get vaccinated. They will also be requested to select a date for their next vaccination. Receipt of each vaccination will be contingent on completion of all the previous doses (i.e. in order to receive the 3rd vaccination participants will need to have first completed the 1st and 2nd vaccinations), with no skipping of doses being allowed. After receiving their vaccinations, participants in the incentivised groups will be provided with the appropriate shopping vouchers. Two days prior to their 2^nd^ and 3^rd^ vaccination sessions, the Healthy Incentives team will send participants text messages reminding them of their appointments.

### Participant recruitment and randomisation

**Figure 1:**
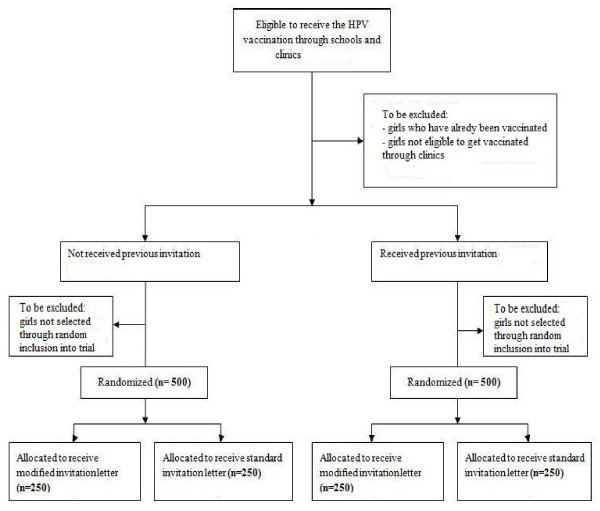
Recruitment and randomisation of participants.

To be included in the study, participants will be selected randomly from a list of names of all girls aged 16-18 years, meeting the above inclusion criteria (See Figure 1). This list will be compiled by the Birmingham Primary Care Shared Services Agency (BPCSSA), which holds and controls all Birmingham patient data, from the names of all 16-18 year old girls eligible to be vaccinated against HPV. The list will be sorted according to whether girls have received a previous invitation to get vaccinated but have failed to attend their first session or have not previously received an invitation. BPCSSA will randomly select 500 participants to be included in the trial from each of these two sub-lists using the RAND() function in Excel. Selected individuals from both the samples will subsequently be separately randomised, via the aforementioned technique, to receive one of two invitations letters:

(a) A standard letter inviting them to attend their first vaccination session (Additional file 1) or

(b) A modified invitation letter, which will include the offer of vouchers worth £45 for receiving the three vaccinations (Additional file 1)

This will result in the groups presented in Table 1

**Table 1:** Incentivised and control groups

	**Receiving invitation for 1**^**st **^**time**	**Having received an invitation previously**
Control Group	250 (receiving standard invitation letters; no incentives)	250 (receiving standard invitation letters; no incentives)
Intervention Group	250 (receiving modified invitation letters; incentives)	250 (receiving modified invitation letters; incentives)

## Outcomes

### Uptake

Uptake of each vaccination by participants will be recorded at the community clinics where vaccinations will take place.

### Social deprivation

Levels of social deprivation will be measured by using participants’ postcodes to calculate Index of Multiple Deprivation (IMD) scores. This is a measure of multiple deprivation measured at the small area level, i.e. the Lower Layer Super Output Area (LSOA). It is made up of seven LSOA level domain indices, which relate to income deprivation, employment deprivation, health deprivation and disability, education skills and training deprivation, barriers to housing and services, living environment deprivation, and crime. IMD scores range from 0 to100, with higher scores indicating higher levels of deprivation.

### Informed choice

In order to assess whether the offer of financial incentives undermines the quality of decisions to undertake the HPV vaccinations, a short modified version of a validated measure of informed choice will be used [16]. This will consist of (Additional file 2):

1. Two items rated on a seven point scale, assessing attitudes towards the HPV vaccination: “For me, having the HPV vaccination is (a) 1: not at all good −7: extremely good and (b) 1: not at all harmful-7: extremely harmful.”

2. Three items assessing knowledge of the HPV vaccination by requesting participants to determine the validity (whether true or false) of three statements relating to the vaccination: “If I have the HPV vaccination: I am less likely to get cervical cancer**;** I am less likely to get other sexually transmitted diseases; I am less likely to get pregnant.”

### Sample size determination

According to the latest report from the Department of Health on coverage of the HPV vaccinations [7], the average completion rate for the “catch-up” campaign targeting females aged 17-18 years in the Birmingham East & North Primary Care Trust is 32.4%. Previous studies investigating the impact of financial incentives on uptake of vaccinations have reported an avergage between-group difference of approximately 8.5%: Specifically, Moran et al. [17] reported an effect size of 8.5% for uptake of the influenza vaccination with incentives (20.3% (control group) vs 28.8% (incentivised group)) and Yokley et al. [18] reported an avergage increase of 8.4% in childhood immunisation across three time points with the addition of inventives (at two weeks: 10.1% (control group) vs. 22.5% (incentivised group); at 2 months: 22.7% (control group) vs. 30.8% (incentivised group); at three months: 26% (control group) vs 30.8% (incentivised group)). Based on these figures, we expect financial incentives in this study to increase completion (i.e. uptake of the 3^rd^ vaccination) of the HPV vaccination programme by 8.5%, resulting in a completion rate of 40.9% by incentivised groups. To detect this difference between arms using a two-tailed *χ*^2^ test at the 5% significane level with 80% power, a sample of 1008 participants is required (calculations performed in GPower 3.0); This figure has been rounded off to the nearest whole number, resulting in a required sample of 1000 participants (half of whom consist of previous-non attenders and half of whom, first-time invitees), giving 500 in each intervention arm (See Table 1).

### Evaluation

The evaluation of the financial incentive scheme will be conducted by researchers at King’s College London, Centre for the Study of Incentives in Health (CSI Health, http://www.kcl.ac.uk/csihealth). Data relating to participants’ uptake of each of the three HPV vaccinations, along with their postcodes, age and answers to the measure of informed choice will be transferred by the Healthy Incentives team to CSI Health researchers. All information will be anonymised and kept securely. Data will be transferred via email in password protected files. CSI Health researchers will analyse the data with the aim of: i) determining the impact of financial incentives on uptake of the HPV vaccination and on the quality of girls’ decisions to get vaccinated; and ii) writing up and publishing the findings.

### Statistical analysis

To assess the impact of the intervention on initial uptake (i.e. the 1st vaccination) and completion of the HPV vaccination programme (i.e. the 3rd vaccination) logistic regressions will be performed separately for each of the two samples, i.e. for girls who have not received an invitation to get vaccinated before and those who have received a previous invitation but have failed to attend. To test the moderating effect of social deprivation on the impact of the intervention, the interaction between IMD scores and intervention will be added to the logistic regression models. To test whether there is a difference in the size of effect of the intervention in the two samples, datasets will be combined and another logistic regression conducted, in which whether participants have received an invitation to get vaccinated before or or not will be added as a predictor to the model, along with the intervention. To test for differences in the attrition rates between the 1^st^ and 3^rd^ vaccinations between the invervention and control groups the *χ*^2^ test will be used. Finally, differences in knowledge of the HPV vaccination between intervention and control group will be tested using the *χ*^2^ test, while differences in attitudes towards the HPV vaccination will be examined via a one-way analysis of variance. All tests will be assessed at the 5% level of significance.

## Discussion

The results of the study will produce valuable information regarding the potential effectiveness of financial incentives in increasing uptake and completion of the HPV vaccinations by teenage girls. The results will also provide valuable information regarding the validity of concerns about the potentially adverse effects of financial incentives on the quality of people’s decisions to engage in incentivised behaviours. If evidence from this trial supports such concerns, further research will be needed to assess how incentives might undermine informed choice, e.g. whether they alter who attends or whether they alter the attitudes towards and/or knowledge of the target behaviour in all who are offered incentives and therefore in those who attend. The design of the present trial does not allow for such assessments to be made.

Knowledge regarding the impact of financial incentives both on uptake of the HPV vaccination and on the quality of decisions to engage in incentivised behaviours is lacking in the literature. Findings therefore, are expected to clarify these issues and have the potential to inform discussions concerning the increasing use of financial incentives for health promotion.

### Research governance

The trial is run by the Birmingham East and North Primary Care Trust, in partnership with the Young Foundation, as part of the former’s service development. In consultation with the Trust, it was deemed that ethical approval was not required for its implementation. Ethical Approval was sought for researchers at King’s College London, Centre for the Study of Incentives in Health, to access data from the Birmingham East and North Primary Care Trust in order to evaluate the financial incentives scheme. This was granted by the Birmingham East and North Research Ethics Committee (reference 11/WM/0073, 8^th^ April 2011). NHS Permission for Research was granted by the Birmingham and the Black Country Comprehensive Local Research Network (BBC CLRN) Research Management & Governance (RM&G) Consortium Office on behalf of the BBC CLRN RM&G Consortium Trusts (reference BENPC040.44791, 1^st^ August 2011**).**

### Trial registration

Current Controlled Trials, ISRCTN52339409.

### Funding

The trial is funded by the Birmingham East and North Primary Care Trust. The evaluation is funded by the Wellcome Trust, as part of a Strategic Award in Biomedical Ethics; programme title: “The Centre for the Study of Incentives in Health”; grant number: 086031/Z/08/Z.

## Competing interests

The authors declare that they have no competing interests.

## Authors’ contributions

TMM advised on the research design. EM drafted the protocol. All authors provided input on the protocol and read and approved the manuscript.

## Pre-publication history

The pre-publication history for this paper can be accessed here:

http://www.biomedcentral.com/1472-6963/12/301/prepub

## Supplementary Material

Additional files 1 HPV Vaccination Invitation Letters. Click here for file

Additional files 2 HPV Vaccination Survey Form. Click here for file
