# Supplementary material for: Using financial incentives to increase initial uptake and completion of HPV vaccinations: protocol for a randomised controlled trial
Source: BMC Health Serv Res. 2012 Sep 4;12:301. doi: 10.1186/1472-6963-12-301 (PMC3471042; doi:10.1186/1472-6963-12-301)
Supplement: Additional files 1 — HPV Vaccination Invitation Letters. [file 1472-6963-12-301-S1.pdf]

Additonal material 1

Standard HPV vaccination Invitation Letter – Control Groups

Birmingham East and North

Heart of Birmingham
Teaching Primary Care Trust

MISS XXXXX XXXXX
ADDRESS LINE 1
ADDRESS LINE 2
ADDRESS LINE 3
POSTCODE

Immunisation Team
Newtown Health Centre
171 Melbourne Avenue
Newtown
Birmingham
B19 2JA

Date

Tel: 0345-245-0777

Dear XXXXXX,

We are stamping down on cancer and inviting you to attend your first Human Papilloma Virus (HPV) vaccination. A leaflet about the vaccination is included and you can call us on the local rate number 0345-245-0777 if you would like further information.

This is being offered to all girls 12 – 18 years of age and provides protection from cervical cancer.

The vaccination will be held at:

Sutton Cottage Hospital
27a Birmingham Road
Sutton Coldfield
West Midlands
B72 1QH on **XX/XX/XXXX** at **XX:XX.**

If you need to arrange a different time or would prefer to attend a different clinic then please call the appointment line on the local rate number 0345-245-0777, Monday to Friday between 9am and 4pm.

Please do not contact the Health Centre directly to rearrange your appointment as they will not be able to help with this.

Yours sincerely

The Immunisation Team

Map

(Photo of map included here)

Modified HPV vaccination Invitation Letter – Intervention Groups

NHS Birmingham East and North | NHS Heart of Birmingham Teaching Primary Care Trust

«PATIENT_TITLE» «PATIENT_FORENAME»
«PATIENT_SURNAME»
«PATIENT_PREMISES»
«PATIENT_ADDRESS_STREET»
«PATIENT_ADDRESS_LOCALITY»
«PATIENT_ADDRESS_TOWN»
«PATIENT_ADDRESS_POST_CODE»

Immunisation Team
Safeguarding Offices
Gee Business Centre
Holborn Hill
Aston
Birmingham
B7 5JE
Tel: 0345-245-0777

Date

Dear «Patient_Title» «PATIENT_SURNAME»,

We are writing to invite you to attend for your first Human Papilloma Virus (HPV) Vaccination.

This is being offered to all girls 12 – 18 years of age and provides protection from cervical cancer. You have been selected for a limited pilot incentive scheme where you will receive Love2Shop vouchers for receiving this vaccination. These can be redeemed at over 80 High Street stores. The vaccination is in 3 stages – you will receive £20 voucher for attending the 1st vaccination, £5 voucher for the 2nd and £20 for the 3rd.

Your appointment to receive the vaccination will be at:

Partners In Health
163 Yardley Green Road
Bordesley Green
Birmingham
B9 5PU on «Current_appointment_date» at «Current_appointment_time».

If you need to arrange a different time or would prefer to attend a different clinic then please call the appointment line on the local rate number 0345-245-0777, Monday to Friday between 9am and 4pm. Please note that in order to receive vouchers you must attend on the date specified above (or a revised mutually agreed date).

Please do not contact the Health Centre directly to rearrange your appointment as they will not be able to help with this.

A leaflet about the vaccination is included and you can call us on the local rate number 0345-245-0777 if you would like further information.

Yours sincerely

The Immunisation Team

Map

(Photo of map included here)
