# Supplementary material for: Using financial incentives to increase initial uptake and completion of HPV vaccinations: protocol for a randomised controlled trial
Source: BMC Health Serv Res. 2012 Sep 4;12:301. doi: 10.1186/1472-6963-12-301 (PMC3471042; doi:10.1186/1472-6963-12-301)
Supplement: Additional files 2 — HPV Vaccination Survey Form. [file 1472-6963-12-301-S2.pdf]

Additonal material 2: Survey

NHS
Birmingham East and North

NHS
Heart of Birmingham
Teaching Primary Care Trust

HPV VACCINATION SURVEY FORM

Name: ______________________

Mobile Number: ______________________

To help us improve our service we are asking all those coming for HPV vaccinations to answer a few questions.

3. For me, having the HPV vaccination is (**please circle your answer**):
   1 2 3 4 5 6 7

   (1 = Not at all good; 7 = Extremely good)

4. For me, having the HPV vaccination is (**please circle your answer**):
   1 2 3 4 5 6 7

   (1 = Not at all harmful; 7 = Harmful)

5. Please state which of the following you think would be true or false if you have the HPV vaccination (**please tick as appropriate**):

	True	False	Don't
I am less likely to get cervical cancer			
I am less likely to get other sexually			

1. So we can increase the HPV vaccination take up rate we'd like to talk in detail to a small group of women about their experiences. These interviews would take place at home sometime over the next few months. They are entirely optional but will help us to improve our services. Would you be happy for us to call you about arranging an interview?
   YES / NO:______

2. We may share some of your protected health information with the third parties who perform services for us necessary to operate the incentive scheme. In those cases we have written agreements with the third parties that they will not use or disclose your information for any other purposes, except as required by law. Your confidentiality and privacy will be protected at all times and will be processed in accordance with the 1988 Data Protection Act. NHS Birmingham East and North will take all reasonably necessary steps to ensure your data is treated securely and confidentially. Additionally, text messages will be sent to you reminding you of your 2nd & 3rd vaccination dates. The information will also be used in writing a research report on how well the healthy incentives scheme helps increase the take up rate of the HPV vaccination – note that no one will be identified by name. We may contact you in the future regarding health related programmes or services. If you do not wish to participate in the scheme at any point, or require further information, you can contact us on 0345-245-0777. Are you happy to take part in the scheme?

   Tick box if you agree to the Terms & Conditions ☐
